# The role of lipooligosaccharide phosphorylcholine in colonization and pathogenesis of *Histophilus somni* in cattle

**DOI:** 10.1186/1297-9716-43-49

**Published:** 2012-06-07

**Affiliations:** 1 Center for Molecular Medicine and Infectious Diseases, Virginia-Maryland Regional College of Veterinary Medicine, Virginia Tech, Blacksburg, VA 24061, USA; 2 Department of Large Animal Clinical Sciences, Virginia-Maryland Regional College of Veterinary Medicine, Virginia Tech, Blacksburg, VA 24061, USA; 3 Edward Via College of Osteopathic Medicine – Virginia Campus, Blacksburg, VA 24060, USA

## Abstract

*Histophilus somni* is a Gram-negative bacterium and member of the Pasteurellaceae that is responsible for respiratory disease and other systemic infections in cattle. One of the bacterium’s virulence factors is antigenic phase variation of its lipooligosaccharide (LOS). LOS antigenic variation may occur through variation in composition or structure of glycoses or their substitutions, such as phosphorylcholine (ChoP). However, the role of ChoP in the pathogenesis of *H. somni* disease has not been established. In *Haemophilus influenzae* ChoP on the LOS binds to platelet activating factor on epithelial cells, promoting bacterial colonization of the host upper respiratory tract. However, ChoP is not expressed in the blood as it also binds C-reactive protein, resulting in complement activation and killing of the bacteria. In order to simulate the susceptibility of calves with suppressed immunity due to stress or previous infection, calves were challenged with bovine herpes virus-1 or dexamethazone 3 days prior to challenge with *H. somni.* Following challenge, expression of ChoP on the LOS of 2 different *H. somni* strains was associated with colonization of the upper respiratory tract. In contrast, lack of ChoP expression was associated with bacteria recovered from systemic sites. Histopathology of cardiac tissue from myocarditis revealed lesions containing bacterial clusters that appeared similar to a biofilm. Furthermore, some respiratory cultures contained substantial numbers of *Pasteurella multocida,* which were not present on preculture screens. Subsequent biofilm experiments have shown that *H. somni* and *P. multocida* grow equally well together in a biofilm, suggesting a commensal relationship may exist between the two species. Our results also showed that ChoP contributed to, but was not required for, adhesion to respiratory epithelial cells. In conclusion, expression of ChoP on *H. somni* LOS contributed to colonization of the bacteria to the host upper respiratory tract, but phase variable loss of ChoP expression may help the bacteria survive systemically.

## Introduction

*Histophilus somni* (*Haemophilus somnus*) is one of the primary pathogens responsible for bovine respiratory disease and may also cause thrombotic meningoencephalitis (TME), myocarditis, polyarthritis, septicemia, and reproductive failure, among other infections. *H. somni* virulence factors include expression of immunoglobulin binding proteins (IgBP) (which has 2 cytotoxic direct repeat Fic domain motifs near the C terminus [1,2]), survival in phagocytic cells [3], induction of apoptosis in endothelial cells [4,5], antigenic phase variation and endotoxic activity of the lipooligosaccharide (LOS) [6,7], and biofilm formation [8]. LOS antigenic variation occurs randomly in vitro or in response to the specific host immune response, and may occur through variation in composition or structure of the LOS [6,7,9]. The LOS may also be modified by the incorporation of terminal sialic acid, which blocks antibody binding to specific epitopes and enhances bacterial resistance to the bactericidal action of normal serum [10]. *H. somni* LOS can also be modified by the variable addition or expression of phosphorylcholine (ChoP) [11,12]. However, the role of ChoP in the pathogenesis of *H. somni* disease has not been established.

The expression of ChoP on the LOS of *Haemophilus influenzae* is subject to phase variation, and expression of ChoP correlates with colonization of the nasopharynx in infant rats [13], chinchillas [14], and humans [15]. Nontypable *H. influenzae* ChoP binds to platelet activating factor receptor (PAF-R) on human epithelial cells, resulting in adhesion to and invasion of those cells. In addition, when *H. influenzae* binds the PAF receptor coupling to a heterotrimeric G protein complex occurs, resulting in a cascade of host cell signaling and bacterial invasion [16,17]. ChoP expression also results in decreasing the immune response to *H. influenzae* LOS in the bacterial biofilm [18] and promotes maturation of the biofilm [19]. Conversely, ChoP binds human C-reactive protein (CRP), which is an acute phase reactant present in the blood. Binding to CRP results in activation of complement through the classical pathway and killing of the bacterial cells [13]. Therefore, expression of ChoP on *H. influenzae* LOS plays a role in colonization of the respiratory tract and turning off such expression promotes systemic dissemination [13].

Expression of ChoP on the LOS of *H. somni* is also subject to phase variation [11,12]. *H. somni* disease is associated with vascular endothelial cell damage and subsequent formation of thrombi in the affected tissues [5,20]. *H. somni* adheres to bovine endothelial cells [21], and bovine platelets activated by *H. somni* can cause apoptosis of those endothelial cells [4,22]. However, the role of PAF-R in adherence to and apoptosis of bovine endothelial cells has not been examined.

In this study, we investigated the role of ChoP expression on *H. somni* LOS in colonization of the bacteria in the bovine respiratory tract and systemic dissemination. We also examined the role of ChoP in adhesion to respiratory epithelial cells. Our results indicated that, like *H. influenzae*, colonization of the bovine respiratory tract by *H. somni* is associated with expression of ChoP, while bacteria that invade systemically are associated with the loss of ChoP expression. Our results also suggest that ChoP contributed to, but was not required for, adhesion to respiratory epithelial cells.

## Materials and Methods

### Bacterial strains and growth conditions

*H. somni* strain 738 has been previously described [23]. *H. somni* strain 7735 and Bovine Herpes Virus type 1 (BHV-1) was kindly provided by Dr Andrew Potter, Veterinary Infectious Disease Organization (VIDO), University of Saskatchewan, Canada. *H. somni* strains were grown on Columbia agar base (Difco culture media, Becton Dickinson and Company, Franklin Lakes, NJ, USA) supplemented with 5% ovine or bovine blood (CBA), and are listed in Table 1. CBA plates were incubated 16–24 h at 37°C in a candle extinction jar or in the presence of 5% CO_2_[24]. For growth in broth medium, a loopfull of multiple colonies was used for inoculation to assure that a random population of cells was selected. The colonies were suspended in phosphate buffered saline, pH 7.2, (PBS) prior to inoculation in brain heart infusion (BHI) broth supplemented with 0.1% Trizma base and 0.01% thiamine mono-phosphate [24]. Stocks of all bacterial strains were maintained at −80°C in 10% skim milk.

**Table 1:** ***H. somni*strains used in this study**

Strain	Source	Reference
738	Clonal isolate of 2336	[25]
738P	ChoP-positive clonal isolate of 738	[11]
738P+	ChoP-positive clone of 738	This work
738P-	ChoP-negative clone of 738	This work
7735	Pneumonic lung isolate	A. Potter, Veterinary Infectious Disease Organization. University of Saskatchewan, Canada
7735R	Streptomycin resistant clone of 7735	This work
7735 + 1	ChoP+ clone of 7735R	This work
7735 + 2	ChoP+ clone of 7735	This work

### Colony immunoblotting

Detection of *H. somni* colonies expressing ChoP was performed as previously described [9]. Briefly, colonies were blotted onto nitrocellulose (NitroBind, GE Osmonics), and dried. The membranes were washed, blocked with 1% skim milk in TBS for 1 h at room temperature, incubated with anti-ChoP monoclonal antibody (MAb) 5-F5.9 [11] or TEPC-15 [26] (Sigma-Aldrich, Saint Louis, MO) overnight at 4°C, and then washed with TBS. Both MAbs have been shown to be specific for ChoP [26]. The membranes were incubated with horse radish peroxidase (HRP) conjugated to anti-mouse IgG or IgA (Jackson Immunoresearch Laboratories) for detection of anti-ChoP MAb 5-F5.9 or TEPC-15, respectively. The membranes were washed with TBS and developed in 4-chloro-1-napthol (BioRad, Hercules, CA) in the presence of 0.015% H_2_O_2_. For selection of ChoP-positive (ChoP+) or negative (ChoP-) clones, single reactive or non-reactive colonies were selected from the CBA plates and subcultured. The colony blotting was then repeated to obtain colonies that were either predominantly positive (65-95%) or negative (> 90%) for ChoP.

### Animal challenges

Six-to 18-week old Holstein male calves were used in three animal challenge experiments to examine the role of ChoP expression in the pathogenesis of *H. somni*. All animals were purchased from local breeders and were determined to be free of respiratory disease and pathogens by clinical examination and culture of nasopharyngeal swabs. Animals from the same experimental group were housed in separate pens with access to separate outdoor holding areas. Animals were fed hay and grain and had access to fresh drinking water *ad libitum*. All animals were examined daily for clinical symptoms during the experiment. Experiment 1 was performed using ChoP+ or ChoP- isolates of strain 738, which is well characterized, particularly in regard to LOS structure [11,23]. ChoP+ or ChoP- isolates of strain 7735, which is capable of causing severe disease in a bovine respiratory disease model (A. Potter. personal communication), were used for experiments 2 and 3.

For Experiment 1, two calves were challenged intranasally (IN) and transtracheally (TT) with strain 738 or a ChoP+ isolate of that strain obtained from a previous study (738P) [11]. Calves were first inoculated with 2 × 10^7^ PFU (plaque forming units) of a low virulence bovine herpes virus-1 (BHV-1) [27] (provided by Dr Andrew Potter, VIDO) IN 3 days before *H. somni* challenge to suppress their innate immune defenses. This strain of BHV-1 is of low virulence and capable of causing an infection in calves, inducing stress and cytokine levels, but does not cause pneumonia or systemic clinical symptoms [27]. *H. somni* strains for challenge were grown in supplemented BHI medium to mid-log phase and washed twice in PCM (PBS containing 0.5 mM MgCl_2_ and 0.15 mM CaCl_2_). The calves were challenged with 5 mL of 6 × 10^9^ colony forming units (CFU)/mL of *H. somni* IN and 10 mL of 1.6 × 10^10^ CFU/mL TT. Calves where challenged IN and TT to promote bacterial colonization of the upper and lower respiratory tracts. Nasal swabs were taken daily post challenge, and trans-tracheal washes (TTW) were performed 24 h and 96 h post challenge to collect bacteria present in the lower respiratory tract. For TTW, the trachea was trocharized and a French catheter was used to inject 50 mL of sterile PCM into the lower respiratory tract. The solution was aspirated into sterile 50-mL conical tubes and stored on ice. Bacteria were sedimented by centrifugation for 20 min at 15 100 × *g*, diluted and spread on CBA plates, and incubated at 37°C in a candle extinction jar. Colony immunoblotting was performed to determine the percentage of colonies that were reactive to anti-ChoP MAb, as described above.

For Experiment 2 strain 7735 was used to assess if expression of ChoP on another strain would yield similar results to those obtained with strain 738. Strain 7735 was passed several times on CBA plates containing incremental amounts of streptomycin until the strain was able to grow on media containing 80 μg/mL of streptomycin. A streptomycin-resistant clone (7735R) and a ChoP+ isolate from that strain (7735 + 1) were each used to challenge a group of four animals. For suppressing innate immunity prior to *H. somni* challenge, each animal was inoculated with BHV-1 virus as described for Experiment 1. In addition, each animal was injected with 0.1 mg/kg/day of the corticosteroid dexamethasone intramuscularly for four days starting three days before *H. somni* challenge to determine if further suppression would enhance colonization or systemic infection. Animals were challenged IN and TT with 5 mL of 6.6 × 10^8^ CFU/mL or TT with 10 mL of 8 × 10^8^ CFU/mL per inoculation route. One TTW was performed 24 h post-challenge and samples were processed as described in Experiment 1. Nasal swabs and blood samples were collected periodically. Blood samples for bacteriological examination were collected in Vacutainer tubes containing sodium polyanethol sulfonate (Becton, Dickinson and Company, Franklin Lakes, NJ, USA) to suppress phagocytosis. A volume of 100 μL was spread on CBA plates, incubated at 37°C under 5% CO_2_, and examined for bacterial growth after 24 and 48 h. TTW, nasal swabs, and blood samples were spread on CBA plates in addition to CBA plates containing 40 μg/mL streptomycin, 3 μg/mL lincomycin, and 5 μg/mL vancomycin to suppress the growth of bacteria other than *H. somni*[28,29]. Two animals from each group were euthanized when no further clinical symptoms were observed, followed by a post-mortem examination. Tissue sections were collected for histopathological examination or processed in a stomacher for 10 min to homogenize the tissues (500 μL PBS/gm) and 70–100 μL were spread on CBA plates. The plates were incubated at 37°C in a candle extinction jar and examined for bacterial growth after 24–48 h.

Experiment 3 was performed using a ChoP+ or ChoP- isolate of *H. somni* strain 7735. The ChoP+ isolate (7735 + 2) and the ChoP- isolate (7735-) were each used to challenge a group of four calves. To minimize the number of *in-vitro* passages, isolates 7735 + 2 and 7735- were selected directly from the original stock of strain 7735 and were not subcultured before use. Nine animals were divided into two experimental groups of four animals each, and one negative control. Two animals from each group were challenged through the IN, TT, and intravenous (IV) routes, and two were challenged only by IN and TT routes with the ChoP+ or ChoP- variants. The IV challenge was added to assure the bacteria of both phenotypes entered the bloodstream and systemic sites. To suppress innate immunity prior to *H. somni* challenge, each animal was injected with dexamethasone only, as described for Experiment 2, as the combination of BHV-1 and dexamethazone did not appear to enhance systemic infection. Challenge isolates were grown on CBA plates overnight and a portion of the colonies were suspended in PCM and washed twice in PCM. The calves were challenged with 5 mL of 2 × 10^9^ CFU/mL IN or 10 mL of 5 × 10^10^ CFU/mL TT and IV per inoculation route. The control animal received similar doses of dexamethasone and was inoculated with sterile physiological saline only through the IN, TT, and IV routes. One TTW was performed 24 h post challenge and processed as described above. All animals were euthanized when no further development of clinical symptoms was observed. Euthanasia was performed as approved by the Institutional Animal Care and Use Committee. Tissue samples were processed as in Experiment 2. Culture plates that contained pure or predominant *H. somni* colonies from tissue samples were used to determine the percentage of colonies that were reactive to anti-ChoP MAb using colony immunoblotting, as described above.

### Tissue culture adherence

A bovine nasal turbinate (BT) cell line (ATCC, Manassas, VA) was grown as described by the supplier. Frozen stocks were diluted in Dulbecco’s modified Eagle’s medium (D-MEM) supplemented with 10% horse serum and allowed to grow on round glass coverslips placed on the bottom of 24-well tissue culture plates. For visualizing bacterial cells, the *Bac*Light Green bacterial stain (Invitrogen, Carlsbad, California) was used according to the manufacturer’s instructions. ChoP+ and ChoP- bacterial cells were suspended in PBS to an equal density at OD_600_. The cells were stained for 15 min, washed twice in PBS by centrifugation at 13 000 × *g* for 10 min, and suspended in D-MEM. Bacterial cells were incubated with BT cells at a multiplicity of infection of 1:100 for one hour at 37°C. The BT cells were washed three times with D-MEM, and the glass coverslips were removed and visualized under an Olympus BX41 fluorescence microscope (Olympus America Inc, Center Valley, PA, USA) at a wavelength of 480 nm. Microscopic fields were observed under a magnification of 1000 X and a score from one to 10 was given to each field according to the density of the bacterial cells on BT cells. The following scoring scale (bacteria/cell) was used; score 1: 1–3 bacteria, score of 2: 4–10 bacteria, score of 3: 11–20 bacteria, score of 4: 21–30 bacteria, score of 5: 31–40 bacteria, score of 6: 41–60 bacteria, score of 7: 61–80 bacteria, score of 8: 81–100 bacteria, score of 9: 101–150 bacteria, score of 10: more than 150 bacteria. The total score reported for each sample was the mean score obtained from the observation of 40 microscopic fields.

### Statistical analyses

The Chi-Square test for 2 × 2 contingency tables was used for statistical analysis of the bovine challenge results. Analysis of Variance (ANOVA) was used for analysis of tissue culture adherence results. All statistical analyses were performed using JMPIN (SAS Institute Inc, Cary, NC, USA).

## Results

### Animal infection and expression of ChoP

All calves challenged with *H. somni* developed fever within 24 h post-challenge, which was maintained for 12–48 h post-challenge and then returned to normal. Due to conservation of animal numbers, controls were not inoculated with killed *H. somni*. However, the fever was likely due to endotoxin release, which is also common in animals given bacterin vaccines. Initial challenge experiments with *H. somni* only resulted in colonization, but not pneumonia or systemic infection. Because most *H. somni* infections result from stress or previous infection, animals were pre-challenged with a low virulence strain of BHV-1 or given dexamethasone 3 days before *H. somni* challenge.

**Figure 1:**
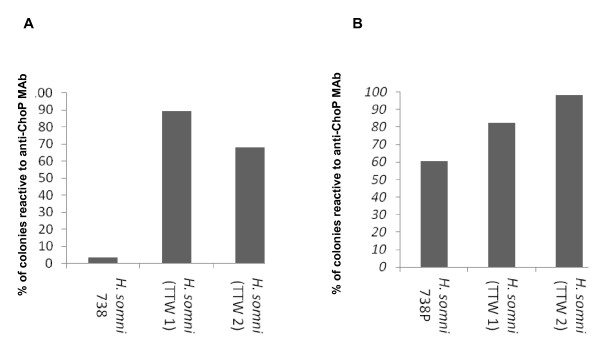
**The percentage of*H. somni*colonies that were ChoP+ from challenge strains and bacteria recovered from the TTW 24 and 96 h post-challenge of calves in Experiment 1.** A: The percentage of ChoP+ colonies from strain 738, which was predominantly ChoP-, and bacteria recovered from the TTW of the calf challenged with that strain at 24 h (TTW 1) and 96 h (TTW 2). B: The percentage of ChoP+ colonies from strain 738P, which was a predominantly ChoP+ isolate of strain 738, and bacteria that were recovered from the TTW of one calf challenged with that strain at 24 h (TTW 1) and 96 h (TTW 2) post-challenge.

Experiment 1: Following culture of respiratory samples, the reactivity of bacterial colonies to anti-ChoP MAb was determined by colony immunoblotting. The total number of colonies examined for each sample varied from 119 to 625 per plate. The percentage of ChoP+ colonies from challenge strain 738 was 3.4%, indicating that the strain was predominantly ChoP-. The percentage of ChoP+ colonies from the TTWs of the calf challenged with that strain was 89.3% at 24 h and 68.1% at 96 h (Figure 1A), resulting in an 85.9% and 64.7% increase in the percentage of ChoP+ colonies compared to the challenge strain at 24 h and 96 h, respectively. The percentage of ChoP + colonies from the TTWs of the calf challenged with isolate 738P (60.5% ChoP+ colonies prior to challenge) was 82.4% and 98.2% at 24 and 96 h, respectively (Figure 1B), resulting in a 21.9% and 37.7% increase in ChoP+ colonies compared to the challenge strain, respectively (*p* < 0.0001). In summary, the population of *H. somni* recovered from the TTW of the calf challenged with predominantly ChoP- strain 738 became predominantly ChoP+, while the population of *H. somni* recovered from the calf challenged with the predominantly ChoP+ isolate 738P remained predominantly ChoP+ (Figure 1).

**Figure 2:**
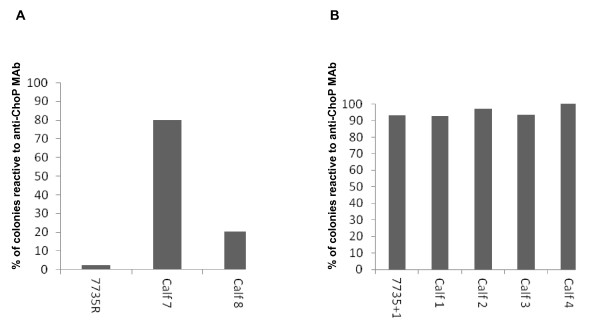
**The percentage of*H. somni*colonies that were ChoP+ from challenge isolates and bacteria recovered from the TTWs of calves in Experiment 2.** A: The percentage of ChoP+ colonies from isolate 7735R, which is predominantly ChoP-, and from *H. somni* recovered from the TTW of the calves challenged with that strain. B: The percentage of ChoP+ colonies from strain 7735 + 1, which is a predominantly ChoP+ derivative of isolate 7735R, and bacteria recovered from the TTW of the calves challenged with that strain.

Experiment 2: Calves 5–8 were challenged with a streptomycin-resistant isolate of *H. somni* strain 7735 (7735R), while calves 1–4 were challenged with a ChoP+ isolate of 7735R (7735 + 1). The total number of colonies recovered from each animal was 70 to 398. The percentage of ChoP+ colonies from isolate 7735R was only 2.5%. The percentage of *H. somni* ChoP+ colonies recovered from the TTW of calve 7 was 80% and from calve 8 was 20.5% (Figure 2A), which was 77.5% and 18% more (*p* < 0.0001) than that of the challenge isolate, respectively. No fluids could be aspirated from the TTW of calves 5 and 6. The percentage of ChoP+ colonies from isolate 7735 + 1, which was used to challenge calves 1, 2, 3, and 4, was 93%. The percentage of *H. somni* ChoP+ colonies recovered from the TTW of calves 1, 2, 3, and 4 was 92.9%, 97.2%, 93.5%, and 100%, respectively (Figure 2B). Thus, the population of 7735 + 1 remained predominantly ChoP+ after recovery from the TTW of these calves (Figure 2B). Bacteria were not recovered from blood samples from any of the calves.

Post-mortem examination of calves 3, 7, and 8 revealed that the calves had varying degrees of purulent bronchopneumonia with abscesses and multifocal purulent myocarditis. Calf 8 had fibrinous polyarthritis that involved the stifle joints. *H. somni* was isolated from the lungs of calf 7, and calf 3 had a myocardial infarction. Histopathological examination of tissue samples revealed that the lungs contained areas of inflammation with infiltration of neutrophils. The lungs also contained multifocal abscess with necrotic purulent centers surrounded by inflammatory cells and fibrin. Heart samples contained multifocal purulent areas with necrotic myofiber centers and infiltration by macrophages, lymphocytes, neutrophils, and some areas of myofiber loss. Vessels in the cardiac muscle had endothelial hypertrophy with fibrin thrombi and purulent inflammation of the vessel walls. Bacterial colonies were observed in heart samples that contained histopathological lesions. *H. somni* was isolated from the heart, kidney, spleen, pericardium, peritoneum, nasopharynx, bronchus, and synovial fluid of different calves. *Pasteurella multocida* was isolated with *H. somni* from many of the samples obtained from internal organs, and therefore the reactivity of isolated *H. somni* to anti-ChoP MAb could not be determined from these samples.

**Figure 3:**
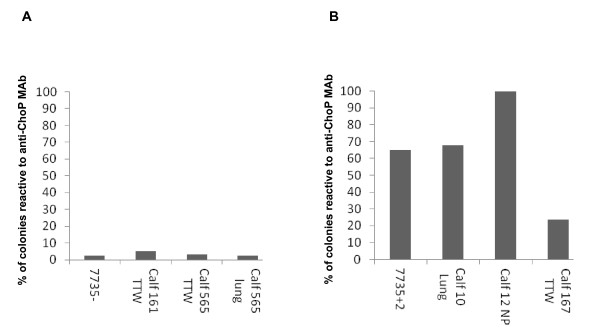
**The percentage of*H. somni*colonies that were ChoP+ from challenge strains and bacteria recovered from the respiratory tracts of calves in Experiment 3.** A: The percentage of ChoP+ colonies from isolate 7735-, which is predominantly ChoP-, and from *H. somni* colonies recovered from the respiratory tract of calves challenged with isolate 7735-. B: The percentage of ChoP+ colonies from isolate 7735 + 2, which is predominantly ChoP+, and from the respiratory tract of calves challenged with that isolate. TTW: Trans-tracheal wash. NP: Nasopharynx.

Experiment 3: Calves 11, 13, 161, and 565 (Group 1) were challenged with a ChoP- isolate of *H. somni* strain 7735 (7735-) and calves 10, 12, 104, and 167 (Group 2) were challenged with a ChoP+ isolate (7735 + 2) that was distinct from the variant used in Experiment 2. Only the TTW of calves 167, 565, and 161 could be examined for reactivity with anti-ChoP MAb. The percentage of ChoP+ *H. somni* colonies from the challenge strains were compared to the percentage of ChoP+ colonies from *H. somni* recovered from the TTW, the lungs, and nasopharynx of challenged calves. The percent of ChoP+ *H. somni* colonies isolated from the lungs of calves 565 and 10, and the nasopharynx of calf 12 were also included (Figure 3).

The percentage of ChoP+ colonies from isolate 7735-, which was used to challenge calves 161 and 565, was 2.5%. The percentage of ChoP+ colonies recovered from the TTW of calf 161 was 5%, and from the TTW and lung of calf 565 was 3.4% and 2.6%, respectively (Figure 3A). The population of *H. somni* used for challenge of calves 161 and 565 was predominantly ChoP- and the population of *H. somni* recovered from the respiratory tract of those calves remained predominantly ChoP- (Figure 3A). The percentage of ChoP+ colonies of isolate 7735 + 2, which was used to challenge calves 10, 12, and 167 was 65%. The percentage of ChoP+ *H. somni* colonies isolated from the lung of calf 10 was 67.9%, from the nasopharynx of calf 12 was 100%, and from the TTW of calf 167 was 23.5% (Figure 3B). The percentage of ChoP+ colonies isolated from the lung of calf 10 and the nasopharynx of calf 12 was higher than that of challenge isolate 7735 + 2 by 2.9% and 35%, respectively. The percentage of ChoP+ colonies recovered from the TTW of calf 167 was less than that of the challenge isolate by 41.5%. The population of *H. somni* used to challenge calves 10 and 12 was predominantly ChoP+ (65%) and the population of *H. somni* recovered from the respiratory tracts of those calves remained predominantly ChoP+ (67.9% and 100%). However, the population of *H. somni* recovered from the TTW of calf 167 was predominantly ChoP- (23.5%) (Figure 3B). No bacteria were isolated from samples obtained from calf 104, and no bacteria were cultured from blood samples collected from any of the calves.

**Figure 4:**
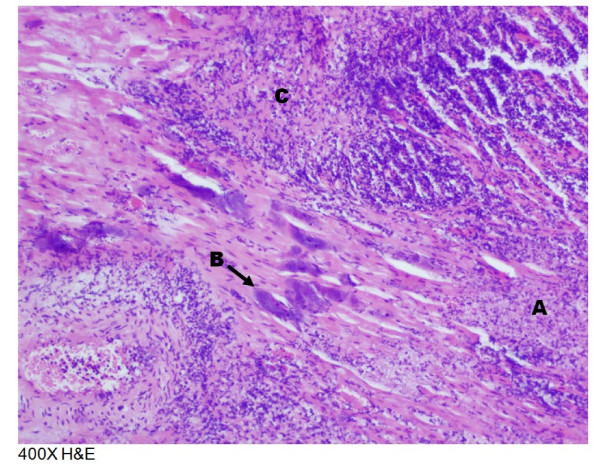
**Photomicrograph of cardiac muscle tissue section from calf 10.** A: A, Myofibrillar necrosis; B, Bacterial clusters; C, Infiltration of lymphocytes and neutrophils.

**Figure 5:**
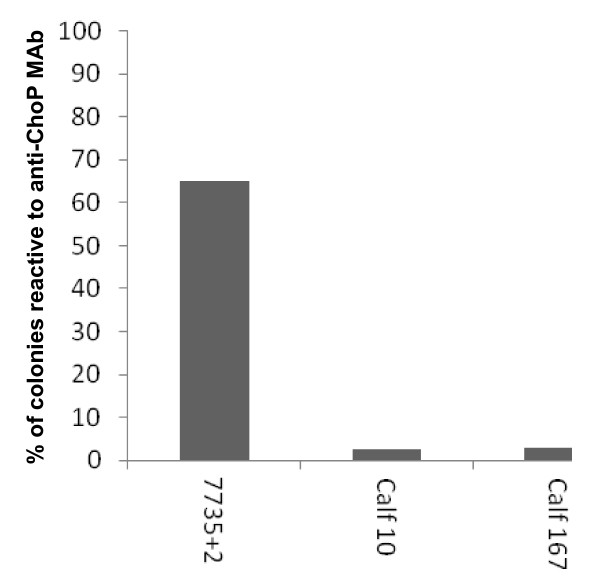
**The percentage of*H. somni*colonies that were ChoP+ from challenge strain 7735 + 2 and bacteria recovered from the hearts of calves in Experiment 3.** The percentage of ChoP+ colonies from 7735 + 2, which is predominantly ChoP+, and the percentage of ChoP+ *H. somni* colonies isolated from the hearts of calves 10 and 167.

Following post mortem examination, two calves in Group 1 had mild bronchopneumonia, one calf had myocardial fibrosis, and one calf had adenitis of the hilar lymph node. Post mortem examination of calves in Group 2 revealed that two calves had bronchopneumonia that varied between mild and purulent, and two calves had myocardial necrosis or fibrosis. Histopathological examination of tissues from Group 1 revealed foci of purulent inflammation with necrosis and neutrophilic infiltration in the liver and foci of kidney inflammation. There was also lymphadenitis of the hilar lymph node with active germinal centers and many neutrophils in addition to inflammation of the airways and alveoli with fibrin. One calf had multifocal suppurative myocarditis with infiltration of macrophages and areas of myofiber loss and replacement with fibrosis. Histopathological examination of tissues from Group 2 revealed foci of inflammation in the liver with necrosis and neutrophilic and lymphocytic infiltration. The hilar lymph nodes were inflamed and had active germinal centers and many neutrophils. The hearts in two calves had multifoci of purulent inflammation with extensive myofiber necrosis and areas of mineralization in addition to areas of fibroplasia, fibrosis, and collagen deposition. The heart lesion shown in Figure 4 contained numerous bacteria in clusters. An enlarged photomicrograph of a lesion from this heart has been published and shown to be bacteria in a biofilm [8]. The lungs of two calves had purulent inflammation in the airways and alveoli with the presence of neutrophils, macrophages, fibrin, and edema. Some bronchioles were surrounded by fibrosis. *H. somni* was isolated from the lungs, bronchi, kidneys, nasopharynxes, spleens, livers, hilar lymph nodes, and hearts of various calves that were challenged. No bacteria were isolated from the internal organs of the control animals. The percentage of ChoP+ *H. somni* colonies from the hearts of calves 10 and 167, which were challenged with isolate 7735 + 2, were 2.73% and 2.76%, respectively, which represents 62.27% and 62.24% fewer ChoP+ colonies, respectively. Thus, the population of the challenge isolate was predominantly ChoP+ while the population of *H. somni* recovered from the hearts of both calves was predominantly ChoP- (Figure 5).

### Adherence of *H. somni* to bovine nasal turbinate cells

**Figure 6:**
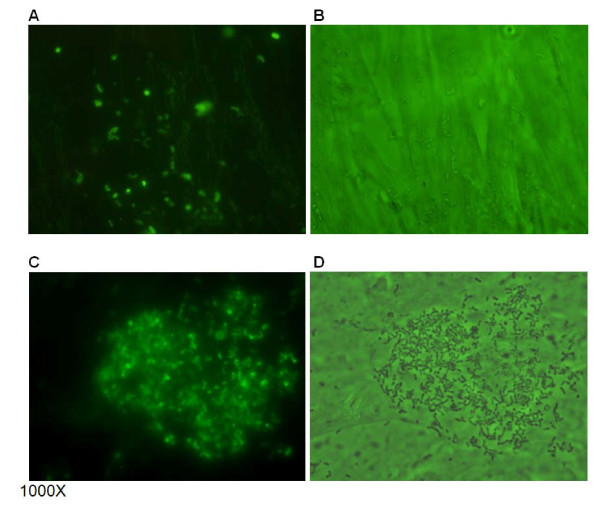
**Photomicrographs of bacterial adherence to bovine turbinate (BT) cells. ChoP+ and ChoP– isolates of*H. somni*strain 738 stained with a fluorescent dye adhered to BT cells.** The adherence was randomly distributed per microscopic field (A and B). However, a large number of *H. somni* 738- were often associated with one particular cell per field (C and D) (cells with more bacteria were more commonly seen). Panels: A, fluorescence microscopy of *H. somni* 738+; B, mixed light and fluorescence microscopy of *H. somni* 738+; C, fluorescence microscopy of *H. somni* 738-; D, mixed light and fluorescence microscopy of *H. somni* 738-.

**Figure 7:**
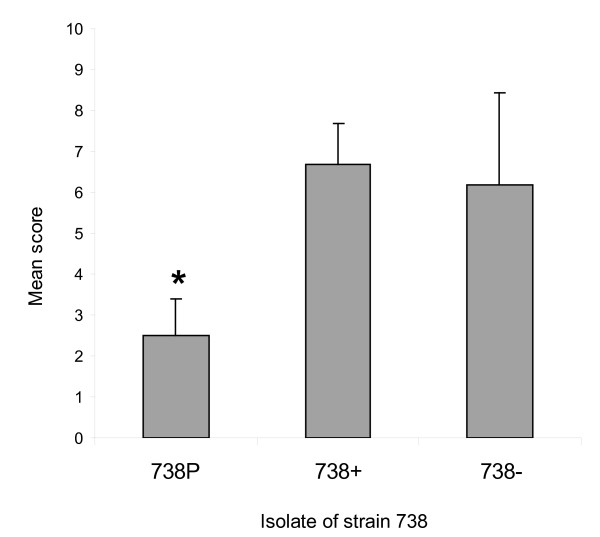
**Adherence of ChoP+ and ChoP- isolates of*H. somni*strain 738 to bovine turbinate (BT) cells.** The mean score for adherence of *H. somni* 738+ and 738- cells to BT cells was similar. However, the mean score for *H. somni* 738P was significantly lower (* = *p* < 0.0001).

*H. somni* 738+ and 738- were examined for adherence to BT cells. In addition, the adherence of isolate 738P, which is a ChoP+ derivative of strain 738 that has a more truncated LOS oligosaccharide [11], was also examined. Bacterial adherence to BT cells was randomly distributed throughout the microscopic fields (Figure 6A and B). However, large numbers of *H. somni* 738- cells were often observed adhering to one particular BT cell in some fields (Figure 6C and D). The mean adherence score for *H. somni* 738+ and 738- cells was similar. However, the mean adherence score for *H. somni* 738P was significantly lower (*p* < 0.0001) (Figure 7).

## Discussion

Phase variation is a common feature of *H. somni* LOS [9]. Although translational phase variation most commonly affects glycosyl transferases that attach sugars to the LOS, we have previously shown that expression of ChoP also occurs due to 5′-AACC-3′microsatellite repeats in the choline kinase gene (*lic1A*)[12]. Furthermore, because ChoP is located on the first glucose adjacent to the inner core oligosaccharide, ChoP is antigenically available in strain 738 only if the outer core is not expressed due to phase variation. Therefore, in strain 738 ChoP must be expressed and the LOS outer core not expressed for ChoP to be accessible to host sites or immunological defenses [12]. Although the phase variation rate of ChoP was not determined, the overall phase variation rate of *H. somni* LOS has been determined to be about 12% [9]. Therefore, selection of particular bacterial LOS phenotypes due to host selective pressure may be high.

More than one factor may play a role in *H. somni* adherence and colonization, including ChoP on the LOS [30] and IgBP [8,31], which has been shown to play a role in *H. somni* biofilm formation [8]. Choline, in the form of phosphatidylcholine, is a component of eukaryotic outer cell membranes. Choline is also found on the outer surface of human and animal pathogens in the form of ChoP. ChoP is incorporated into the cell envelope of *Streptococcus pneumoniae*[32-34], on the LOS of *H. influenzae*[26], on the LOS and pili of *Neisseria* species [35], on the lipopolysaccharide (LPS) of *Aggregatibacter actinomycetemcomitans*[38], on a 43 kDa protein in *Pseudomonas aeruginosa*[36], and on the LOS of *P. multocida*[39]. Among bacteria isolated from the human upper respiratory tract, 15% contained ChoP [40]. Expression of ChoP on *H. influenzae* LOS correlates with colonization of the nasopharynx in an infant rat model [13] and in humans [15]. *H. influenzae* recovered from the lungs of challenged mice has a greater proportion of ChoP+ cells, while ChoP- cells are cleared more rapidly [19]. However, investigating the role of ChoP expression on *H. influenzae* has been hampered by the limitations of experimenting with humans as the natural host. In this study we demonstrate a correlation between expression of *H. somni* ChoP and bacterial colonization of the bovine respiratory tract.

Initial challenge of healthy calves in the respiratory tract resulted in colonization, but not infection (pneumonia and systemic spread to other organs). *H. somni* is an opportunistic pathogen, normally infecting animals that have been compromised by previous infection, stress, weather, etc. Therefore, to simulate the condition of animals susceptible to *H. somni* infection, the animals were either first treated with dexamethasone and/or challenged with an attenuated strain of BHV-1 3 days prior to challenge with *H. somni*. Both treatments were designed to suppress innate and cellular immunity, and were successful in enhancing the susceptibility of the animals to *H. somni* infection and disease. However, the combination of dexamethazone and BHV-1 did not appear to further enhance susceptibility. Animal infection Experiments 1 and 2 indicated there was an enrichment of ChoP+ *H. somni* in the bovine upper respiratory tract, supporting a role for *H. somni* ChoP to act as a ligand in bacterial adherence to epithelial cells of the upper respiratory tract. In Experiment 3 one sample from the respiratory tract of a calf challenged with the predominantly ChoP+ population of *H. somni* had a population that contained less ChoP+ cells than the challenge strain. However, samples recovered from the respiratory tract of other animals in the same group had more ChoP+ cells than the challenge strain and were predominantly ChoP+. In contrast, the bacteria recovered from the upper and lower respiratory tract of calves challenged in experiment 3 with a predominantly ChoP- population of *H. somni* remained predominantly ChoP-. The absence of enrichment of ChoP+ *H. somni* in the upper respiratory tract of some animals may have been due to use of an *H. somni* variant (7735-) that was different from that used in Experiments 1 and 2. Variant 7735- may express unknown surface components that were selected for instead of ChoP during the colonization process leading to obscuring the role of ChoP during colonization. In addition, variant 7735- was derived from strain 7735, but the predominantly ChoP- challenge strains used in Experiments 1 and 2 were parent strains and not ChoP- derivatives of a parent strain. Variant 7735- may also be less capable of varying expression of ChoP than the parent strain, which may have resulted in reduction in the number of cells in the challenge population that were capable of ChoP expression during infection. In addition, co-infection with another bacterial pathogen that competed for ChoP receptors may have occurred. For example, *P. multocida* was often isolated in high numbers in samples with *H. somni*, even though *P. multocida* was not isolated from prechallenge respiratory samples. Subsequent experiments have shown that *P. multocida* forms an excellent biofilm when mixed with *H. somni*, but *Mannheimia haemolytica* does not (I. Sandal et al., unpublished data). Therefore, modulation of ChoP expression or access to receptors may be influenced by co-growth of other bacteria. In addition, we found myocarditis with predominately ChoP+ bacteria a prominent finding in systemically infected calves. Myocarditis is now recognized as a common finding in animals with *H. somni* pneumonia and other systemic infections [41]. Furthermore, *H. somni* biofilms have been shown to be particularly prominent in cardiac tissue of myocarditis cases [8]. The presence of ChoP on the LOS on nontypable *H. influenzae* has been shown to reduce the host inflammatory response and promote formation of stable biofilms [42]. ChoP may serve a similar function in *H. somni*.

ChoP expression on *H. influenzae* LOS assists in adherence and invasion of *H. influenzae* to human bronchial cells through interaction with PAF-R [16]. In addition, *H. influenzae* binding to PAF-R is associated with initiating host cell signaling pathways [17]. PAF-R is present on epithelial and endothelial cells and on blood platelets, and plays a role in the adhesion and invasion of a variety of pathogens [16,38]. Previously, we demonstrated that *H. somni* induced aggregation of bovine platelets, and that this aggregation was through binding of ChoP to PAF-R present on the platelets [30]. Therefore, ChoP on the LOS may contribute to the pathogenesis of *H. somni* through the interaction with PAF-R.

Expression of ChoP on *H. influenzae* LOS is associated with increased susceptibility to killing by normal human serum in the presence of the acute phase reactant C-reactive protein (CRP) [13]. CRP is present in the blood and its expression increases during acute infections. CRP binds ChoP on *H. influenzae* LOS leading to activation of complement through the classical pathway and killing of the bacteria. Therefore, in the absence of ChoP expression *H. influenzae* may be more serum-resistant and capable of systemic invasion. However, the expression of ChoP on *H. influenzae* isolated from systemic locations in humans has not been investigated. In our study we found that *H. somni* recovered from the internal organs of calves challenged with a predominantly ChoP+ population of *H. somni* were predominantly ChoP-, indicating that systemic invasion of *H. somni* was associated with loss of reactivity with anti-ChoP MAb. *H. somni* was not isolated from the internal organs of calves challenged with a predominantly ChoP- population of *H. somni*, except for one sample from which only a few colonies were isolated. This finding may indicate that the predominantly ChoP- population may have had a reduced ability to disseminate systemically due to a reduced ability to colonize, or that the rate of phase transition from ChoP- to ChoP+ in *H. somni* is not as great as it is in *H. influenzae*.

Colonization of the respiratory tract is one of the initial steps in pathogenesis [20,43] and may also be required for systemic invasion. Both ChoP+ and ChoP- populations of *H. somni* were equally capable of adhering to bovine turbinate (BT) cells, indicating that ChoP is not required for adherence to these cells. The diminished ability of *H. somni* isolate 738P to adhere to BT cells may be due to the absence of surface structures on that isolate that allow for efficient binding. Isolate 738P is a ChoP+ isolate obtained from a previous study [11] and has an LOS that is more truncated than the other two isolates used in the BT adherence experiment. Therefore, glycoses or other structures besides ChoP on the outer core of the LOS of isolates 738+ and 738- may play a role in adhesion. LPS has also been shown to contribute to the adherence of *Actinobacillus pleuropneumoniae* to host cells [44]. However, later studies did not find the O-antigen of *A. pleuropneumoniae* LPS contributed to adherence and that bacteria with a truncated O-antigen were more adherent than the parent to porcine lung epithelial cells [45]. An alternative explanation for the equivalent adherence of ChoP+ and ChoP- isolates to BT cells is that the eukaryotic cells may not have a receptor for ChoP, but other surface structures shared by both *H. somni* isolates may allow for equal adhesion to these cells. *H. somni* expresses IgBPs [31], that also act as heparin binding proteins [46,47]. Heparin binding proteins are involved in adherence to endothelial cells [48] and competition assays have demonstrated that the proteins play a role in *H. somni* adhesion to bovine pulmonary [47] and brain [46] endothelial cells. Therefore, IgBP may play a substantial role in the adhesion of both 738+ and 738- cells to BT cells, thereby masking the role of ChoP in adhesion. Thus, our results indicate that *H. somni* colonization of the bovine respiratory tract is associated with expression of ChoP, but that other factors on *H. somni* may play an important role in host colonization.

Clinical manifestations and pathological findings in the challenged calves were less severe than expected. Nonetheless, the pathological lesions in these calves were consistent with reports of *H. somni* infection [20,49-54]. Bronchopneumonia was observed in the majority of tested calves. However, myocarditis was observed in six out of 12 calves and *H. somni* was isolated in large numbers from most of the samples. These results support reports that *H. somni* may be an important cause of myocarditis associated with septicemia [54], and that *H. somni* has been more frequently isolated from necropsy samples that included examination of the heart [53-55]. The presence of what appeared to be a biofilm in the myocardium of the infected calves has been described in more detail [8]. The increase in cases of myocarditis associated with *H. somni* infection may be due to closer attention to the heart during necropsy in cases of pneumonia or TME [54], and is now being diagnosed more often than TME [53]. Clinical manifestations of arthritis were not severe in the calves challenged in our experiments. However, *H. somni* was isolated from the synovial fluid of one calf presenting with severe arthritis. This finding was particularly interesting, as isolation of the organism from synovial fluids or joint samples is uncommon.

In conclusion, our results indicated that ChoP expression was associated with colonization of the respiratory tract of compromised calves, and that loss of ChoP expression was associated with systemic dissemination. Our results also indicated that expression of ChoP was not required for adherence to bovine nasal turbinate cells, but may play a role in the pathogenesis of *H. somni* through binding to PAF-R [30]. Further research is required to determine the identity and contribution of other bacterial factors in colonization of the host.

## Abbreviations

TME: Thrombotic meningoencephalitis; IgBP: Immunoglobulin binding proteins; LOS: Lipooligosaccharide; ChoP: Phosphorylcholine; PAF-R: Platelet activating factor receptor; CRP: C-reactive protein; CBA: Columbia agar base supplemented with 5% ovine or bovine blood; PBS: Phosphate buffered saline, pH 7.2; BHI: Brain heart infusion medium; CFU: Colony forming units; MAb: Monoclonal antibody; HRP: Horse radish peroxidase; ChoP+: ChoP-positive; ChoP-: Cho-P negative; VIDO: Veterinary Infectious Disease Organization; IN: Intranasally; TT: Transtracheally; 738P: Cho-P+ clone of H. somni strain 738; PFU: Plaque forming units; BHV-1: Bovine herpes virus 1; PCM: PBS containing 0.5 mM MgCl2 and 0.15 mM CaCl2; CFU: Colony forming units; TTW: Trans-tracheal washes; 7735R: Streptomycin-resistant clone of H. somni strain 7735; 7735 + 1: ChoP+ isolate from strain 7735R; 7735 + 2: ChoP+ isolate of H. somni strain 7735; 7735-: ChoP- isolate of H. somni strain 7735; IV: Intravenous; BT: Bovine nasal turbinate cell line; D-MEM: Dulbecco’s modified Eagle’s medium; ANOVA: Analysis of Variance.

## Competing interests

The authors declare that they have no competing interests.

## Authors’ contributions

SFE carried out the molecular studies, participated in all animal studies and drafted the manuscript, WKS performed the animal challenges, and TJI designed the experiments and edited and completed the manuscript. All authors read and approved the manuscript.
